# Platelet Microvesicles, Inflammation, and Coagulation Markers: A Pilot Study

**DOI:** 10.3390/hematolrep15040069

**Published:** 2023-12-04

**Authors:** Antonio Gidaro, Alessandro Palmerio Delitala, Roberto Manetti, Sonia Caccia, Mark J. Soloski, Giorgio Lambertenghi Deliliers, Dante Castro, Mattia Donadoni, Arianna Bartoli, Giuseppe Sanna, Luigi Bergamaschini, Roberto Castelli

**Affiliations:** 1 Department of Biomedical and Clinical Sciences Luigi Sacco, Luigi Sacco Hospital, University of Milan, Via G.B. Grassi N° 74, 20157 Milan, Italy; sonia.caccia@unimi.it (S.C.); donadoni.mattia@asst-fbf-sacco.it (M.D.); arianna.bartoli@unimi.it (A.B.); luigi.bergamaschini@unimi.it (L.B.); 2 Department of Medicine, Surgery and Pharmacy University of Sassari, Via San Pietro 43, 07100 Sassari, Italy; aledelitala@uniss.it (A.P.D.); rmanetti@uniss.it (R.M.); d.castro@studenti.uniss.it (D.C.); g.sanna7@studenti.uniss.it (G.S.); 3 Division of Rheumatology, Johns Hopkins University School of Medicine, Baltimore, MD 21224, USA; mski@jhmi.edu; 4 Fondazione Mattarelli Largo della Crocetta, N° 2, 20122 Milan, Italy; giorgio.lambertenghi@unimi.it

**Keywords:** microvesicles, microparticles, C-reactive protein (CRP), Interleukin 6 (IL-6), Interleukin 10 (IL-10), Interleukin 17 (IL-17), transforming growth factor β (TGF-β), fibrinogen, plasminogen activator inhibitor-1 (PAI-1), von Willebrand factor (VWF), homocysteine, thrombin activatable fibrinolysis inhibitor (TAFI), factor VII (FVII), Protein S

## Abstract

Background: Platelet “Microvesicles” (MVs) are studied for their role in blood coagulation and inflammation. The study aimed to establish if MVs are related to age, plasma levels of inflammation, coagulation, and fibrinolysis markers in healthy individuals. Methods: We prospectively enrolled volunteers aged over 18 years. MVs, plasma levels of C-reactive protein (CRP), Interleukin 6 (IL-6), Interleukin 10 (IL-10), Interleukin 17 (IL-17), and transforming growth factor β (TGF-β), fibrinogen, plasminogen activator inhibitor-1 (PAI-1), von Willebrand factor (VWF), homocysteine, factor VII (FVII), thrombin activatable fibrinolysis inhibitor (TAFI), and Protein S were tested. Results: A total of 246 individuals (median age 65 years (“IQR”54–72)) were evaluated. Both univariate analysis and logistic regression models showed that MVs positively correlate with age, CRP, IL-6, IL-10, IL-17, TGF-β, fibrinogen, PAI-1, VWF, FVII, and homocysteine, while inversely correlating with TAFI and Protein S. The ROC curve analysis performed to identify a cut off for MV values (700 kMP) showed a good accuracy with over-range cytokines fibrinolysis factor and coagulation markers. Conclusions: To the best of our knowledge, this study is the first to correlate MVs with an entire panel of cardiovascular risk factors in healthy individuals. A future possible role of MVs in screening exams is suggested.

## 1. Introduction

Initially ectosome or shedding “Micro vesicles” (MVs), also known as microparticles (MPs), were reported as “dust” originating from platelets [1], and their role in blood coagulation was suggested [2]. Plasma membrane generate MVs by the outward budding and shedding and their diameter ranges from 50 to 1000 nm. This process is not unique to platelets but affects many other cells lines. MVs were originally thought to be a mechanism for maintaining cellular homeostasis to eliminate unnecessary material. However, it is now thought that MVs play a role in intercellular communication [3,4]. MVs are present in the blood of healthy individuals, representing important mediators in maintaining tissue homeostasis as intercellular communicators. Giesen et al. [5] demonstrated for the first time that neutrophils and monocytes secrete tissue factor (TF), activating a coagulation cascade which is released in circulating blood and can be found in large amounts on MVs near the surface of platelets [5]. Although platelets play a key role in coagulation and clot formation, the clotting ability of platelet MVs is thought to be 50- to 100-fold higher than that of triggered platelets [5], indicating a central role of platelet MVs in blood coagulation. TF activates factor FVII to FVIII, and this process represents the beginning of the extrinsic coagulation process. In addition, the negatively charged surface of MVs catalyzes FIX and FVIII.

In the literature, high levels of MVs are described in several cardiovascular diseases, including metabolic syndrome [6], coronary heart disease [7], atrial fibrillation [8], vascular stent implantation [9], unprovoked deep vein thrombosis [10], and acute pulmonary embolism (PE) [11].

For example, patients with atherosclerosis plaques have higher platelet MV values than individuals without atherosclerosis, even after statistical correction for cardiovascular risk factors [7]. Again, higher platelet MV levels have been found during the acute phase of atherosclerotic stroke [9]. Lastly, elevated levels of platelet MVs have been associated with prolonged injury after myocardial infarction (MI) [7].

Even though the direct involvement of platelet MVs in tumor pathogenesis has not yet been established, some papers have suggested an association between platelet MVs and malignancy [12]. For instance, the total number of platelet MVs was higher in multiple myeloma active treatment patients compared with “watch and wait” management in smoldering multiple myeloma [12].

Moreover, numerous papers have determined that platelet MV levels are increased during various autoimmune diseases [13]. Some authors have pointed out that platelet MVs are localized in the joints of patients affected by autoimmune disease, where they act with an autoimmune mechanism, leading to inflammation. This is the case of citrullinated fibrinogen and vimentin, and the formation of platelet–MV–autoantibody complexes proved to be highly effective in triggering the formation of pro-inflammatory leukotrienes from neutrophils [13]. The evaluation of platelet MV levels in rheumatic diseases has been the subject of several papers. Taken together, these works suggest that platelet MV levels are altered in autoimmune diseases and that an increase in their heterogeneity may reveal biomarkers for these syndromes. Yet, as of today, the literature does not unanimously address the MV as a biomarker or consequence of disease.

Several studies correlate high MV levels with a single cytokine [14,15,16] or coagulation proteins [17,18,19,20,21,22,23,24,25,26,27,28,29,30,31,32,33,34,35,36]. They include in vitro and in vivo studies using human samples and animal models. So far, however, no studies have been conducted to correlate MV levels with multiple coagulation proteins and cytokines in the human plasma of healthy individuals in order to explore a possible role of MV in a screening exam.

Therefore, the aim of this study is to establish if MVs are related to the age and plasma levels of factors controlling inflammation (in particular, plasma levels of C-reactive protein (CRP), Interleukin 6 (IL-6), Interleukin 10 (IL-10), Interleukin 17 (IL-17), and transforming growth factor β (TGF-β)), coagulation (von Willebrand factor (VWF), fibrinogen, plasminogen activator inhibitor-1 (PAI-1), factor VII (FVII), homocysteine), and fibrinolysis (thrombin activatable fibrinolysis inhibitor (TAFI), and Protein S).

## 2. Materials and Methods

### 2.1. Study Population

From 2 July 2013 to 5 May 2020, we prospectively enrolled healthy individuals over 18 years old. All the volunteers were blood donors under 65 years or previous blood donors that were recalled. The volunteers were defined as “Healthy” and included in the study if the exclusion criteria where respected. In particular, volunteers were excluded in the following cases: (1) they have ongoing treatment with anti-coagulant, anti-platelet, anti-diabetic drugs, or anti-fibrinolytic or anti-hypertensive agents; (2) they have used anti-inflammatory drugs in the 3 months before sample collection; (3) they have active or previous smoking activity, chronic lung illness (i.e., ASMA; COPD), renal failure, previous or active auto-inflammatory diseases, or cancers; (4) C-reactive Protein (CRP) >10 mg/L at blood exam evaluation.

All the patients who were included in the study were followed-up over time to document the incidence of venous thromboembolism (VTE).

The local ethical committee permitted the study (Ospedale Maggiore Policlinico di Milano; N 206 date 29 June 2013), and all trial volunteers provided a written informed consent.

### 2.2. Sample Collection and Storage

Blood samples were collected after 12 h of fasting. Using sodium citrate 3.8% as an anti-coagulant, antecubital venous blood samples were drawn from patients. Plasma was obtained by centrifuging the samples at 2000× *g* for 20 min at room temperature, which were divided into small aliquots and stored at −80 °C until testing.

### 2.3. Measurements

Via a commercial coagulometric method (Diagnostica Stago, Asnières, France) with intra- and inter-assay CVs of 4% and 7% respectively, fibrinogen was measured. The fibrinogen normal range was 200–400 mg/dL. PAI-1 activity was determined using a commercial bioimmunoassay (Chromolize PAI-1, Bio pool, Umea, Sweden) with intra- and inter-assay CVs of 2.4% and 4.5%. PAI-1 normal range was 5–40 UI/mL. VWF antigen was measured in citrated plasma by an “in-house” sandwich ELISA using two monoclonal antibodies directed against different VWF epitopes (11B6.18 and 7G10.8) [37]. The intra- and inter-assay CVs were both 8%. VWF normal range was 60–160%. FVII activity was dosed using a commercially available one-stage prothrombin time-based assay (Instrumentation Laboratory Company, Lexington, MA, USA) in accordance with the manufacturer’s instructions. The intra- and inter-assay CVs were 10%. FVII normal range was 65–140%. Total homocysteine values were determined via high-performance liquid chromatography (HPLC) with fluorometric detection and isocratic elution. Homocysteine normal range was 4–14 µmol/L. TAFI antigen levels were determined via a sandwich-type ELISA using a monoclonal capturing antibody and a polyclonal detection antibody. TAFI normal range was 75–275%. Free Protein S antigen was measured using the Asserachrom Free Protein S assay (Stago), a one-step ELISA assay that uses 2 monoclonal antibodies specific to distinct epitopes of the free form of Protein S to directly measure free Protein S in plasma. Protein S normal range was 60–130%. CRP was measured via ELISA (Zymutest CRP, Hyphen BioMed, Andresy, France) with intra- and inter-assay coefficients of variation (CVs) of 7–11%. CRP normal range was 0.5–10 mg/L. IL-10 (normal range: 2.6–20 pg/mL), IL-17 (normal range: 1.3–20 pg/mL), and TGF-β (normal range: 10–2500 pg/mL) were measured using Sandwich ELISA immunoassays (Milliplex Human Cytokine, Chemokine Assay, Millipore-Sigma Merck and MILLIPLEX MAP TGF-β Magnetic Bead Single Plex Kit—Immunology Multiplex Assay Merck).

### 2.4. Microvesicles Determination

MV levels were analyzed in platelet-free plasma (whole blood was first centrifuged at 2500× *g* for 15 min to obtain platelet-poor plasma; subsequently, the platelet-poor plasma was transferred to a new centrifugation tube and centrifuged at 20,000× *g* for 30 min to obtain essentially platelet-free plasma). MV characterization was performed according to the MISEV2018 guidelines [38]. Plasma samples were stained with the following antibodies: CD41a-FITC (BD Pharmingen), annexinV-PE (BioLegend, San Diego, CA, USA), CD45-BrilliantViolet421 (BioLegend), and CD144-PE/Cy7 (BioLegend) (1 µL of each per 50 µL of plasma) for 15 min at 37 °C. They were then fixed with PFA and diluted with PBS directly before the measurement. PFA and PBS were filtered through a 0.1 µm filter to reduce the background. Samples were measured on a NovoCyte Quanteon Flow Cytometer (Agilent, Santa Clara, CA, USA) at a slow flow rate with no gating for a fixed amount of time. The MV unit measure is kMP, equal to the MV count/μL.

### 2.5. Statistical Analysis

We performed a Kolmogorov–Smirnov test to evaluate the normal distribution of data. Quantitative data were presented as median values and interquartile ranges (25th and 75th percentiles) due to skewed distribution. Univariate analysis was performed to assess the relationship between MVs and each cytokine and coagulation parameter and to establish the better scatter line (linear, quadratic, or cubic).

A linear regression model (if all scatter lines were linear) or polynomial model (if at least one scatter line were quadratic or cubic) were used for assessing the correlation between MVs (dependent variable) and age and the parameters that resulted from univariate analysis (independent variables) in the whole population enrolled. Pseudo R2 for linear logistic regression model was calculated, and a *p*-value inferior to 0.05 was considered statistically significant. For the multivariate analysis, a *p*-value inferior to 0.05 was considered statistically significant, and for each parameter, the expected B value was calculated. Two different models were evaluated, one for MVs adjusted for age and cytokines (CRP, IL-6, IL-10, IL-17, and TGF-β), and in the second model, we tested MVs adjusted for age and coagulation parameters (fibrinogen, PAI-1, VWF, homocysteine, FVII, TAFI, and Protein S).

We performed an ROC curve analysis to identify a cut off for MV values that correlate with over-range plasma levels of fibrinogen, PAI-1, VWF, homocysteine, FVII, CRP, IL-6, IL-10, IL-17, and TGF-β and under-range values of TAFI, Protein S.

Lastly, we performed Mann–Whitney test (for non-parametric data) to compare groups. A *p*-value inferior to 0.05 was considered statistically significant. Data were analyzed using the SPSS PC statistical package, version 17.00 (SPSS Inc., Chicago, IL, USA).

## 3. Results

During the observational period, 1460 volunteers were evaluated, but only 246 were negative with respect to the exclusion criteria and thus included in the study, with a median age of 65 years (IQR 54–72). A total of 85 subjects were females and 161 were males (Table 1).

In the univariate analysis, MV levels positively correlated with age and levels of fibrinogen, PAI-1, vWF, F VII, and homocysteine, while they were inversely correlated with levels of TAFI and Protein S. In our analysis, MV did not correlate to sex (*p* = 0.447), and for this reason, gender was not included in the multivariate analysis.

With regard to inflammation markers, MV levels positively correlated with levels of CRP, IL-6, IL-10, IL-17, and TGF-β.

All of the better scatter lines observed were linear, and, for this reason, a linear regression model was performed.

The linear regression model confirmed a significant correlation with age, fibrinogen, PAI-1, vWF, FVII, homocysteine, and MVs. The negative correlation of MVs with TAFI and Protein S was also confirmed by the model. The pseudo R2 multiple of the logistic regression model was 0.856 (*p*-value <0.001) (Table 2).

The linear regression model confirmed a significant correlation with IL-6, IL-10, IL-17, TGF-β, and MVs. Both the correlations between CRP and age with respect to MVs were not confirmed in the multivariate analysis. The pseudo R2 multiple of the linear regression model was 0.861 (*p*-value < 0.001) (Table 3).

**Figure 1:**
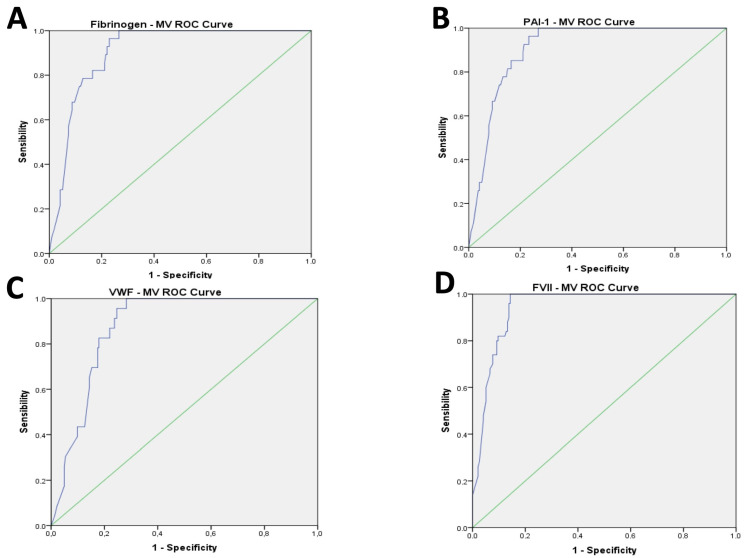

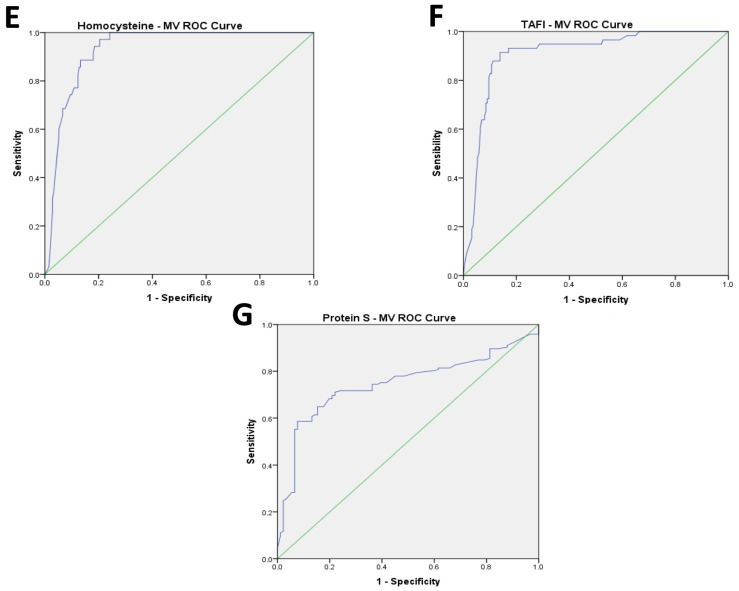
(**A**–**G**): Results of AUC of ROC curve analysis for coagulation parameters.

The AUC for the ROC curve analysis performed to identify a cut off for MV values showed a good accuracy with over-range plasma levels of fibrinogen (AUC: 0.908; CI 0.868 to 0.947, *p* < 0.001), PAI-1 (AUC: 0.908; CI 0.869 to 0.947, *p* < 0.001), VWF (AUC: 0.876; CI 0.83 to 0.922, *p* < 0.001), homocysteine (AUC: 0.908; CI 0.865 to 0.951, *p* < 0.001), FVII (AUC: 0.944; CI 0.917 to 0.971, *p* < 0.001), and even for the under-range value of TAFI (AUC: 0.930; CI 0.898 to 0.962, *p* < 0.001) and Protein S (AUC: 0.729; CI 0.658 to 0.801, *p* < 0.001) (Figure 1A–G).

The cut-off of 700 kMP MVs, chosen based on the ROC curve analysis, showed good sensitivity (SE) and specificity (SP) in predicting the high value of fibrinogen (SE = 0.946; SP = 0.771), PAI-1 (SE = 0.963; SP = 0.767), VWF (SE = 0.957; SP = 0.753), homocysteine (SE = 0.879; SP = 0.862), and FVII (SE = 0.980; SP = 0.857), and the low value of TAFI (SE = 0.971; SP = 0.796) and Protein S (SE = 0.591; SP = 0.934) (Figure 1A–G).

**Figure 2:**
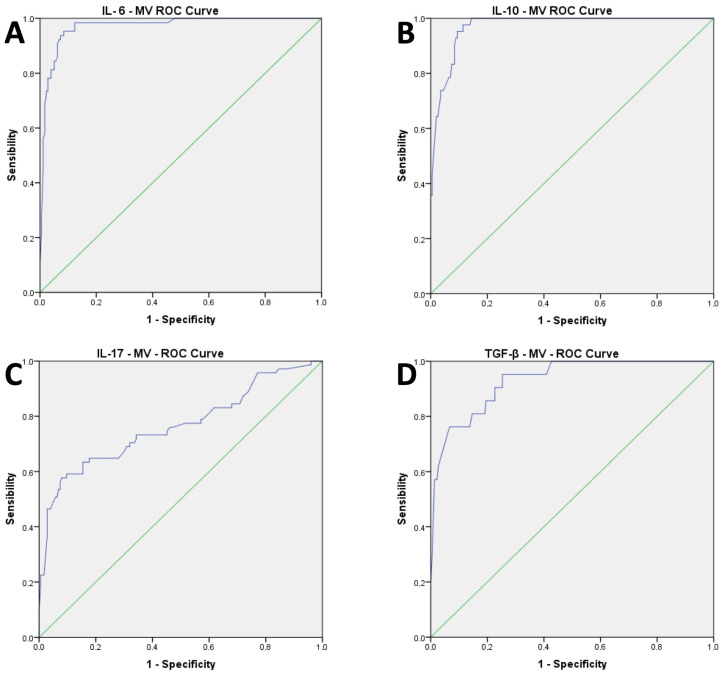
(**A**–**D**)**:** Results of AUC of ROC curve analysis for cytokines parameters.

The AUC for the ROC curve analysis performed to identify the cut-off for MV values showed a good accuracy with over-range plasma levels of IL-6 (AUC: 0.971; CI 0.951 to 0.9992, *p* < 0.001), IL-10 (AUC: 0.972; CI 0.954 to 0.989, *p* < 0.001), IL-17 (AUC: 0.765; CI 0.692 to 0.838, *p* < 0.001), TGF-β (AUC: 0.931; CI 0.881 to 0.981, *p* < 0.001) (Figure 2A–D).

The cut-off of 700 kMP MVs showed good sensitivity (SE) and specificity (SP) in predicting the high value of IL-6 (SE = 0.953; SPE = 0.916), IL-10 (SE = 1; SPE = 0.837), IL-17 (SE = 0.648; SPE = 0.823), and TGF-β (SE = 0.905; SPE = 0.747) (Figure 2A–D).

A total of 77 individuals had MV levels over the cut-off value (700 kMP). Comparing those individuals with the group under 700 kMP, a higher value of CRP (*p* < 0.001), fibrinogen (*p* < 0.001), vWF (*p* < 0.001), PAI-1 (*p* < 0.001), homocysteine (*p* < 0.001), FVII (*p* < 0.001), IL-6 (*p* < 0.001), IL-10 (*p* < 0.001), IL-17 (*p* < 0.001), and TGF-β (*p* < 0.001) was found. on the other hand, a lower value of TAFI (*p* < 0.001) and Protein S (*p* < 0.001) was observed.

During the study, we observed three VTE events. These events have all been detected in the subgroup with MVs greater than 700 kMP. Comparing VTE incidence in the two subgroups, we found that the number of VTE events was statistically higher in patients with MV levels greater than 700 kMP.

## 4. Discussion

In this prospective study, we evaluated the correlation between MVs and age, CRP, fibrinogen, PAI-1, VWF, FVII, homocysteine, TAFI, and Protein S in 246 healthy individuals with a median age of 65 years.

We found that MVs positively correlate with age, CRP, fibrinogen, PAI-1, VWF, FVII, and homocysteine, while inversely correlating with TAFI and Protein S in both univariate and linear regression model analysis. One possible explanation might be that blood coagulation could be activated via MVs and FVII/tissue factor complex, a strong extrinsic coagulation activator [21]. Due to the presence of the procoagulant protein tissue factor (TF) and negatively charged phospholipids such as phosphatidylserine, the surface of MVs can be highly procoagulant. MVs could potentially be implicated in both increased risk of venous thromboembolism and cardiovascular ischemic disease [22].

Several case-control studies have reported increased plasma MV concentrations in patients with arterial or venous thrombosis compared with control subjects [6,7,8,9,10,11,12]. However, conventional retrospective case-control studies are not suitable to evaluate whether the elevated plasma levels of a biomarker are due to disease (reverse causality) or an actual risk factor. In a recent prospective study by Snir et al. [39], the plasma levels of MVs were found to be associated with future risk of venous thromboembolism (VTE), independent of potential confounding factors. This result suggests that an elevated plasma MV level represent a risk marker for VTE events or for rendering atherosclerosis plaque instable.

Our finding of higher number of VTE events in patients with MV levels over 700 kMP is consistent with Snir et al.’s findings [39], which show the link between blood coagulation activation and the risk of thrombosis.

So far, no studies have been conducted which simultaneously measure multiple coagulation proteins in the human plasma of healthy individuals and correlate these with MV levels. Our study, combining multiple measurements and applying univariate analysis and logistic regression models, filled this knowledge gap and both confirms and extends the previous work.

Although this is an observational study with a limited number of healthy volunteers, in light of the data collected, we propose a possible use of MVs in a screening exam. This is due to the observed high SE value obtained in all the ROC curves performed when comparing MVs to fibrinolysis factor and coagulation markers. In particular, we suggest that MV levels over 700 kMP also include the evaluation of fibrinogen, PAI-1, VWF, homocysteine, FVII, TAFI, and Protein S levels as well.

This hypothesis could be effective, with a high benefit/cost ratio in patients over 65 in particular, in whom the incidence of cardiovascular disease increases. In these patients, the early initiation of pharmaceutical treatment could reduce cardiovascular mortality and morbidity.

A second scenario is represented by cancer’s associated thrombosis, where high levels of MV could be used to identify those who would benefit from a prophylactic drug in order to reduce VTE development.

Lastly, autoimmune diseases are a well-known thrombotic risk factor [40]. MV use could induce clinicians to obtain a tight follow-up regarding thrombotic risk in patients with high levels.

Currently, many scientists are focusing their attention on MVs, performing a “liquid biopsy” which, with some limitations, can be a substitute for the classic biopsy [41].

The tight correlation between age and MVs was already reported by Gustafson et al. [42] in a younger and smaller cohort. The novelty of our study is the combined correlation between age, MVs, and pro-coagulant markers that explain the well-known age-related higher risk of thrombosis [43,44], as well as the increased cardiovascular risk due to aging.

Key regulators of MV generation in age-related cardiovascular disease seem to be Thrombin and PAI-1. PAI-1 expression is not only increased in old age but also significantly induced in a number of diseases linked with the senescence process [45]. Increased levels of PAI-1 and its pro-coagulant function have been documented as hallmarks of endothelial dysfunction in vascular aging. Increased assays of PAI-1 during aging might induce the beginning of MV formation, followed by increased pro-coagulant activity and thrombin formation [23].

Increased oxidative stress and inflammatory cytokines should be considered the main mechanisms mediating these changes [46].

Our data show that in both univariate analysis and logistic regression models, MV levels positively correlated with pro-inflammatory cytokines (TGF-β, IL-6, IL-17).

It is now generally accepted that platelets are effector cells of the immune system, which is reflected in the roles of platelet MVs, as they can transduce receptors, increase the expression of adhesion proteins on recipient cells, and carry or stimulate the release of cytokines. Indeed, many studies found that platelets MVs stimulate endothelial cells to release cytokines [14,15].

The correlation observed in our study between TGF-β levels and MV confirms the results of previous studies which show a high level of TGF-β in sufferers of VTE [10]. In our study, we observed a clear relationship between increased MVs and TGF-β, confirming a previous study by Yamaguchi et al. [47] which demonstrated that the TGFβ1/SMAD/plasminogen activator inhibitor-1 signaling pathway stimulates the release of tissue factor-bearing microvesicles.

This data support the hypothesis that MVs regulate endothelial cells’ function and afflict both the coagulation system and cytokine release.

We should acknowledge that MV dosage is limited by the presence of many confounders, as reported in the MISEV2028 guidelines [38]. In our study, we tried to reduce this bias (i.e., blood samples were collected while fasting in order to reduce bias due to food intake), but we could not rule out that some of them may have influenced our results.

Finally, we cannot rule out the possible influence of a different process (e.g., difference in centrifugation speed, time) in preparing blood samples with respect to inflammation, coagulation, and fibrinolysis markers and MVs.

## 5. Conclusions

This study is the first to correlate multiple coagulation proteins and cytokines to MVs in a healthy population. We report that an over-range value of MVs correlates with a reduction of the fibrinolysis factor and an increase in coagulation markers. We believe these findings will be informative for future studies on MVs aiming to explore their role as a cardiovascular risk factor and useful biomarker.

In addition, considering that the TF/FVII complex has a key role in the activation of the coagulation cascade, new drugs targeting this complex could potentially be challenging to counteract the hypercoagulable state linked with aging.

## Figures and Tables

**Table 1:** Statistical analysis of microvesicles (MVs), age, gender, and coagulation markers (plasma levels of CRP, fibrinogen, PAI-1, vWF, homocysteine, FVII, TAFI, and Protein S) in whole population (A); individuals with MV levels under 700 kMP (B); individuals with MV levels over 700 kMP (C). Data are reported as “median (IQR)” (white lines) or absolute numbers (percentage) (gray lines).

	A. Whole Population	B. Individuals with MVs ≤ 700 kMP	C. Individuals with MVs > 700 kMP	*p*-Value B vs. C
Number of patients	246	169	77	
Age (years)	65 (54–72)	61 (48–68)	72 (70–73)	<0.001
Gender male/female	161/85	136/73	25/12	0.85
Body mass index (BMI)	23.7 (21.5–24.8)	23.5 (21.4–24.6)	23.8 (21.6–24.9)	0.85
Median follow-up (months)	40.1 (27–51)	40.2 (28–51)	39.9 (27–49)	0.64
Venous thromboembolism	3/246	0/169	3/77	0.03
CRP (mg/L) 0.5–10 mg/L	6 (3–12)	4 (2.3–8)	7 (5–9)	<0.001
Fibrinogen (mg/dL) 200–400 mg/dL	262 (117–321)	134 (109–256)	340 (300–435)	<0.001
Fibrinogen > 400 mg/dL	28	1 (3.6%)	27 (96.4%)	<0.001
PAI-1 (UI/mL) 5–40 UI/mL	20.0 (12.0–26.25)	15 (4–20)	36 (25–46)	<0.001
PAI-1 > 40 UI/mL	27	1 (3.7%)	26 (96.3%)	<0.001
vWF (%) 60–160%	68 (62–120)	64 (58–68)	140 (111–164)	<0.001
vWF > 160%	23	1 (4.4%)	22 (95.6%)	<0.001
FVII (%) 65–140%	67 (52–110)	54 (40–67)	150 (120–160)	<0.001
F VII > 140%	50	1 (2%)	49 (98%)	<0.001
Homocysteine (µmol/L) 4–14 µmol/L	8 (4–14)	5 (3–6)	18 (14–22)	<0.001
Homocysteine > 14 µmol/L	58	7 (12.1%)	51 (87.9%)	<0.001
TAFI (%) 75–275%	117 (80–132)	127 (115–138)	78 (66–84)	<0.001
TAFI < 75%	35	1 (2.9%)	34 (97.1%)	<0.001
Protein S (%) 60–130%	56 (44–67)	65 (46–68)	46 (43–48)	<0.001
Protein S <60%	115	47 (40.9%)	68 (59.1%)	<0.001
IL6 (pg/mL) 0.64–10 pg/mL	2 (4–12)	4 (2–5)	20 (12–30)	<0.001
IL6 (pg/mL) > 10 pg/mL	64	4 (6.3%)	60 (93.7%)	<0.001
IL10 (pg/mL) 2.6–20 pg/mL	12 (12–18)	12 (12–12)	22 (18–26)	<0.001
IL10 (pg/mL) > 20 pg/mL	42	0	42 (100%)	<0.001
IL17 (pg/mL) 1.3–20 pg/mL	20 (18–22)	20 (18–20)	22 (20–24)	<0.001
IL17 (pg/mL) > 20 pg/mL	71	26 (36.7%)	45 (63.3%)	<0.001
TGF-β (pg/mL) 10–2500 pg/mL	367 (311–2348)	321 (307–498)	2424 (2280–2512)	<0.001
TGFβ (pg/mL) > 2500 pg/mL	21	2 (9.6%)	19 (90.4%)	<0.001

**Table 2:** Result of univariate analysis (A: *p*-value; B: Pearson correlation) and linear regression model analysis for coagulation parameters (C: *p*-value; D: B value (95% CI)).

	A: Univariate Analysis *p*-Value	B: Univariate Analysis Pearson Correlation	C: Linear Regression Model *p*-Value	D: B (95% CI)
Age (year)	<0.001	0.574	0.008	3.23 (0.84–5.62)
Fibrinogen (mg/dL)	<0.001	0.647	0.019	0.4 (0.07–0.74)
PAI-1 (UI/mL)	<0.001	0.683	0.002	4.16 (1.5–6.82)
vWF (%)	<0.001	0.834	0.018	1.91 (0.34–3.48)
F VII (%)	<0.001	0.744	0.001	1.47 (0.64–2.3)
Homocysteine (µmol/L)	<0.001	0.639	<0.001	6.76 (3.36–10.16)
TAFI (%)	<0.001	−0.69	<0.001	−3.06 (−4.22–−1.89)
Protein S (%)	<0.001	−0.451	≤0.001	−4.21 (−6.16–−2.27)

**Table 3:** Result of univariate analysis (A: *p*-value; B: Pearson correlation) and linear regression model analysis for cytokines (C: *p*-value; D: B value (95% CI)).

	A: Univariate Analysis *p*-Value	B: Univariate Analysis Pearson Correlation	C: Linear Regression Model *p*-Value	D: B (95% CI)
Age (year)	<0.001	0.574	0.195	1.33 (−0.69–3.34)
CRP (mg/dL)	<0.001	0.406	0.514	1.54 (−6.48–3.41)
TGF-β (pg/mL)	<0.001	0.843	>0.001	0.141 (0.1–0.18)
IL-6 (pg/mL)	<0.001	0.850	>0.001	14.3 (9.6–19)
IL-10 (pg/mL)	<0.001	0.816	>0.001	21.6 (15.5–27.8)
IL-17 (pg/mL)	<0.001	0.456	0.02	13.3 (2.1–24.5)

## Data Availability

The study data will be made available upon request to the corresponding author.
